# Platelet-Derived Growth Factor Receptor β Is Critical for Zebrafish Intersegmental Vessel Formation

**DOI:** 10.1371/journal.pone.0011324

**Published:** 2010-06-25

**Authors:** Katie M. Wiens, Hyuna L. Lee, Hiroyuki Shimada, Anthony E. Metcalf, Michael Y. Chao, Ching-Ling Lien

**Affiliations:** 1 Department of Surgery, Keck School of Medicine, University of Southern California and The Saban Research Institute of Childrens Hospital Los Angeles, Los Angeles, California, United States of America; 2 Department of Pathology, Keck School of Medicine, University of Southern California and The Saban Research Institute of Childrens Hospital Los Angeles, Los Angeles, California, United States of America; 3 Department of Biology, California State University San Bernardino, San Bernardino, California, United States of America; Harvard University, United States of America

## Abstract

**Background:**

Platelet-derived growth factor receptor β (PDGFRβ) is a tyrosine kinase receptor known to affect vascular development. The zebrafish is an excellent model to study specific regulators of vascular development, yet the role of PDGF signaling has not been determined in early zebrafish embryos. Furthermore, vascular mural cells, in which PDGFRβ functions cell autonomously in other systems, have not been identified in zebrafish embryos younger than 72 hours post fertilization.

**Methodology/Principal Findings:**

In order to investigate the role of PDGFRβ in zebrafish vascular development, we cloned the highly conserved zebrafish homolog of PDGFRβ. We found that *pdgfrβ* is expressed in the hypochord, a developmental structure that is immediately dorsal to the dorsal aorta and potentially regulates blood vessel development in the zebrafish. Using a PDGFR tyrosine kinase inhibitor, a morpholino oligonucleotide specific to PDGFRβ, and a dominant negative PDGFRβ transgenic line, we found that PDGFRβ is necessary for angiogenesis of the intersegmental vessels.

**Significance/Conclusion:**

Our data provide the first evidence that PDGFRβ signaling is required for zebrafish angiogenesis. We propose a novel mechanism for zebrafish PDGFRβ signaling that regulates vascular angiogenesis in the absence of mural cells.

## Introduction

The development of blood vessels occurs in two distinct stages. Vasculogenesis is defined as the formation of new blood vessels resulting from angioblast aggregation followed by the lumenization of vascular endothelial cells. Angiogenesis is a secondary process to vasculogenesis and occurs via the sprouting of blood vessels from pre-existing vascular structures [1], [2]. During blood vessel development, endothelial cells initially fuse together to form the vascular lumen. Supporting mural cells are then recruited to endothelial cells to stabilize the blood vessel wall and to aid in the formation of the extracellular matrix [3].

The regulation of vasculogenesis and angiogenesis each involves multiple cell types and signaling molecules necessary to coordinate the formation of the vascular system. Platelet-derived growth factor (PDGF) activates a specific family of receptor tyrosine kinases and is involved in the development of blood vessels in chicks and mammals [4]. PDGF-A and PDGF-B ligands can form homo- or heterodimers that bind to the PDGF receptors (PDGFR) α or β. PDGF-B and PDGFRβ null mice die late in embryonic development with renal and cardiovascular abnormalities and fatal hemorrhages [5], [6], [7]. Tissue-specific knockouts show that PDGF-B is secreted by endothelial cells and acts to recruit mural cells (smooth muscle cells and pericytes) for vascular support [8]. Thus, PDGF-B and PDGFRβ paracrine signaling drives the recruitment of smooth muscle and pericyte progenitor cells to the wall of new blood vessels [9], [10], [11], [12].

The zebrafish embryo is an excellent model to use for investigating vascular development. The transparency of zebrafish embryos allows for convenient observation during development while transgenic lines that express fluorescent tags in endothelial cells facilitate the study of developing blood vessels [13], [14]. Zebrafish vasculogenesis and angiogenesis are two distinct vascular processes and occur at different phases of vascular development. During zebrafish development, the *de novo* formation of the dorsal aorta and the posterior cardinal vein of the tail occurs via the fusion of angioblast precursor cells and is considered vasculogenesis. The subsequent sprouting and extension of the intersegmental vessels (ISVs) from the dorsal aorta is considered angiogenesis [15]. More recently, it was shown that formation of the posterior cardinal vein occurs via sprouting and segregation from the dorsal aorta in a process that is distinct from vasculogenesis or angiogenesis [16].

Here we provide the first analysis of PDGF signaling in vascular development in the zebrafish. We found that PDGFRβ is expressed adjacent to the dorsal aorta in the hypochord as early as 20 hours post fertilization (hpf). At these early stages of zebrafish development, there is no evidence of the presence of mural cells in the vasculature, although primitive mural cell markers are present around the anterior portion of the dorsal aorta beginning at 72 hpf [17]. Inhibition of PDGFR signaling using a PDGFR tyrosine kinase inhibitor caused a decrease in the number and extent of ISV formation. Further, morpholino knockdown specific to PDGFRβ and a dominant negative PDGFRβ transgenic line both demonstrated that PDGFRβ is required for angiogenesis of the ISVs. Our results suggest a new role for PDGFRβ signaling in zebrafish vascular development that functions in the absence of mural cells during early angiogenesis.

## Results

### Zebrafish PDGF-B and PDGFRβ were highly conserved with other species

**Figure 1:**
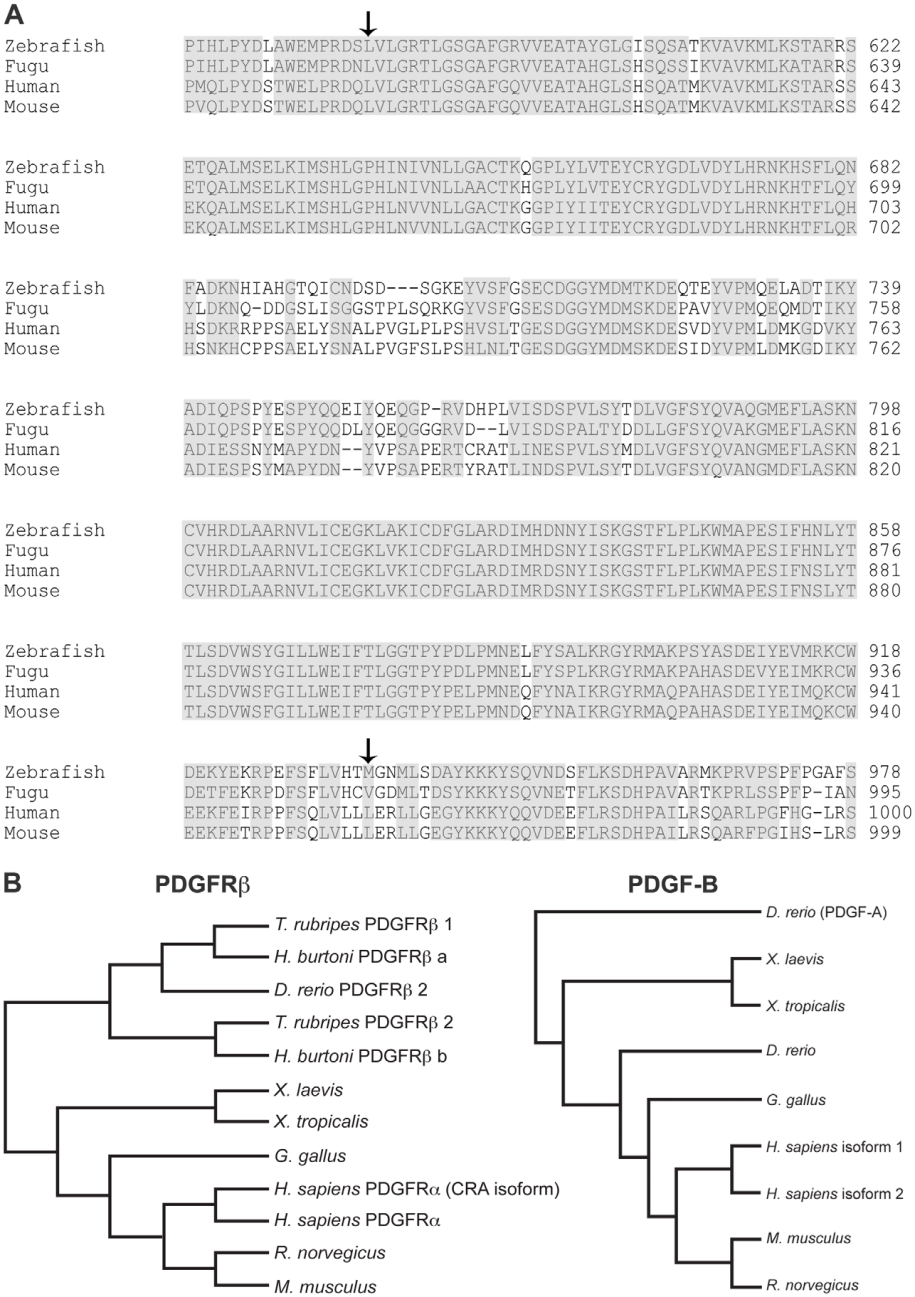
Sequence and phylogenetic analysis of PDGFRβ. Alignment of the zebrafish PDGFRβ tyrosine kinase domain with known PDGFRβ proteins of human, mouse, and *Fugu* (A). Phylogenetic analysis of PDGFRβ and PDGF-B proteins (B). Arrows in A indicate the beginning and end of the tyrosine kinase domains of PDGFRβ.

We previously cloned and characterized a gene encoding zebrafish PDGF-B [18], and recently cloned the zebrafish homolog of PDGFRβ2 (Accession No. HM439112) which is syntenic to *colony stimulating factor 1 receptor* on chromosome 14. Comparison of zebrafish PDGFRβ2 with human, mouse, *Fugu* and other species indicated that the predicted tyrosine kinase domain of zebrafish PDGFRβ2 was highly conserved across multiple species (Figure 1A). Zebrafish PDGFRβ2 was more similar to *Fugu* PDGFRβ1 than *Fugu* PDGFRβ2. Phylogenetic analysis on predicted PDGF-B and PDGFRβ amino acid sequences indicated that both proteins were closely related to their respective vertebrate homologs (Figure 1B). The grouping of amino acid sequences was consistent with the phylogeny of the major vertebrate groups, strongly suggesting that the genes arose early in vertebrate evolution. The phylogenetic analyses of PDGFRβ isoforms using the entire amino acid sequence strongly supported a gene duplication within the represented ray-finned fishes, with all nodes supported at 100%. A second analysis including the zebrafish PDGFRβ1 was consistent with this finding, with the 64 amino acid fragment clustering with the corresponding *D. rerio* PDGFRβ2 fragment. This result suggested a more recent duplication of isoforms within zebrafish. However, the bootstrap support for the groupings within fish taxa for PDGFRβ1 was relatively low (54–69%), therefore this tree is not presented. These analyses also indicated that zebrafish PDGF-B is evolutionarily distinct from PDGF-A. Both PDGF-B and PDGFRβ are thus highly conserved members of their respective protein families (Figure 1).

### Expression patterns of *pdgfrβ* and *pdgf-b* suggested a role for PDGF signaling in developing zebrafish blood vessels

**Figure 2:**
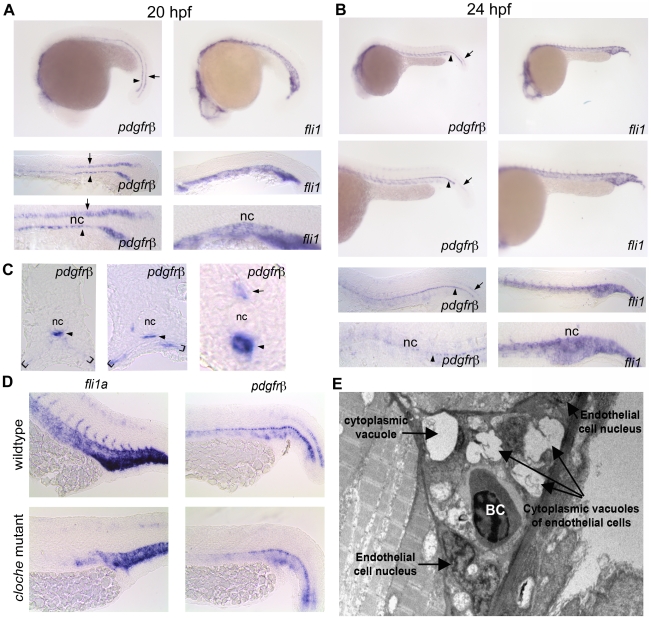
*pdgfrβ2* expression in the developing zebrafish. *In situ* hybridization of embryos at 20 hpf (A) and 24 hpf (B) using an anti-sense probe against *fli1a* or *pdgfrβ2*. *pdgfrβ2* was expressed in the floorplate (arrow) and the hypochord (arrowhead) at 20 hpf. *fli1a* is known to be expressed in the tail vasculature. Floorplate expression of *pdgfrβ2* diminished by 24 hpf while expression in the hypochord persisted and spread ventrally in association with the dorsal aorta and posterior cardinal vein (B). Cross section analysis further indicated *pdgfrβ2* expression in the hypochord (arrowhead), floorplate (arrow) and ventral somite boundary (brackets). *cloche* mutant embryos showed normal expression of *pdgfrβ2* indicating that *pdgfrβ2* was not expressed in endothelial cells of the dorsal aorta or posterior cardinal vein (D). Transmission electron micrograph images of a longitudinal section through the ISVs (E). Mural cells surrounding the endothelial cells of the ISVs were absent at 72 hpf. Notochord (nc), blood cell (BC).

Using *in situ* hybridization, we found that *pdgfrβ2* was expressed in the floor plate and hypochord at 20 hpf (Figure 2A). At 24 hpf, there was decreased expression in the floor plate while hypochord expression persisted with spreading ventral expression lateral to the dorsal aorta and posterior cardinal vein to the ventral somite boundary (Figure 2B and C). Comparison with *fli1a*, which codes for an endothelial transcription factor, suggested a close association of *pdgfrβ2* to the developing vasculature. However, *pdgfrβ2* was still expressed in *cloche* mutants, which lack endothelial cells [19] (Figure 2D), indicating that *pdgfrβ2* was not expressed in endothelial cells. Cross sections of these embryos at 24 hpf further confirmed expression of *pdgfrβ2* in the hypochord and ventral somite boundary (Figure 2C). The localization of *pdgfrβ2* to the hypochord, adjacent to the dorsal aorta, suggested that *pdgfrβ2* may play an important role in zebrafish vascular development. Vascular endothelial growth factor C (VEGFC), a factor known to affect segmental artery formation, is similarly expressed in the hypochord [20].

A recent report showed the presence of mural cells in the anterior portion of the dorsal aorta and to a lesser extent in the posterior cardinal vein starting at 72 hpf [17]. Although pericytes and smooth muscle cells express PDGFRβ2 in other model systems, these data suggest that zebrafish PDGFRβ2 is likely expressed in other cells associated with the developing vasculature. We further characterized the cellular structure of the developing zebrafish ISVs to rule out the presence of mural cells in these vessels. We performed electron microscopy on longitudinal sections through the ISVs of zebrafish embryos at 72 hpf to examine the ultra structure of developing zebrafish ISVs. We found that mural cells, which typically surround mature blood vessels, were absent in the ISVs at 72 hpf (Figure 2E). This absence of vascular support cells in the ISVs at 72 hpf suggested that the developing blood vessels are very immature and consist mainly of endothelial cells.

**Figure 3:**
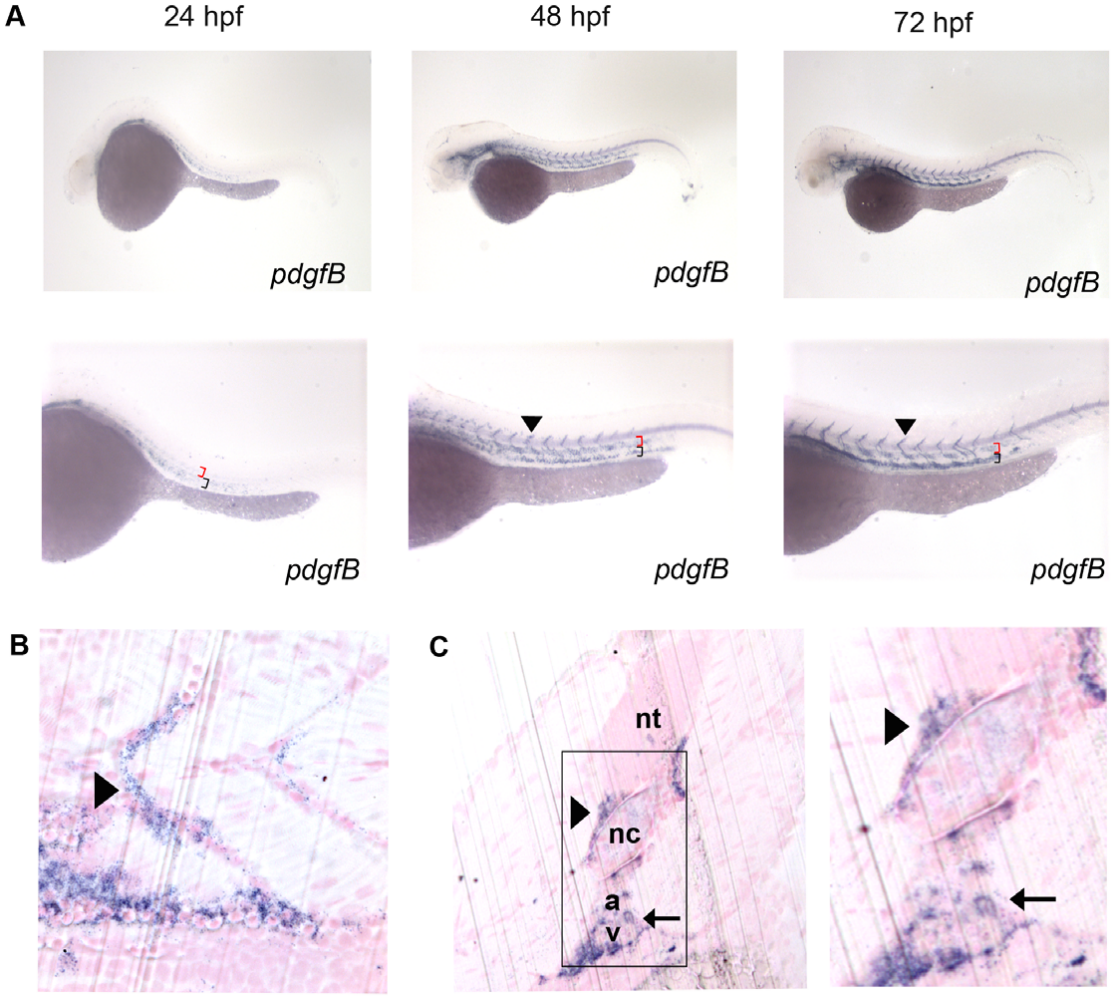
Characterization of *pdgf*-*b* in the developing zebrafish embryo. *In situ* hybridization of embryos at 24, 48, and 72 hpf (A) using an anti-sense probe against *pdgf-b*. *pdgf-b* was expressed at all time points in the head and tail vasculature (arrowheads indicate *pdgf-b* staining in ISVs, red brackets indicate the dorsal aorta, and black brackets indicate the posterior cardinal vein in A). Sagittal (B) and transverse (C) JB-4 sections of 72 hpf embryos analyzed with whole mount *in situ* hybridization using a probe against *pdgf-b. pdgf-b* expression was localized near the dorsal aorta, posterior cardinal vein and ISVs (arrow in C indicates *pdgf-b* staining in dorsal aorta and posterior cardinal vein while the arrowhead indicates staining in the ISV). Neural tube (nt), notochord (nc), dorsal aorta (a), posterior cardinal vein (v).

In mammals, PDGF-B is secreted by endothelial tip cells and is responsible for the proliferation of vascular smooth muscle cells and pericytes [10], [11], [21]. In adult zebrafish, *pdgf-b* expression is upregulated in the regenerating heart and may promote revascularization of the regenerating heart [18]. To determine the expression pattern of *pdgf-b* in the developing zebrafish embryo, we used *in situ* hybridization at 24, 48, and 72 hpf. *pdgf-b* expression was present throughout these stages of development and was localized to the head, dorsal aorta, posterior cardinal vein and ISVs (Figure 3A). The expression pattern was similar to that of mammalian *pdgf-b*, which is expressed mainly in endothelial cells. Sagittal sections of 72 hpf embryos after whole mount *in situ* hybridization showed that *pdgf-b* is localized to the dorsal aorta and ISVs (Figure 3B). Transverse sections further showed that *pdgf-b* expression was localized to the dorsal aorta, the posterior cardinal vein, and the ISVs (Figure 3C). Thus, *pdgf-b* and *pdgfrβ2* are both likely candidates for determining the structure and function of the developing zebrafish vasculature.

### PDGFR inhibition decreased the number and extent of ISV formation

To determine the function of PDGFR signaling in zebrafish blood vessel development, we blocked PDGFR signaling using a tyrosine kinase inhibitor selective to PDGFRs. *kdrl:GFP* [22] transgenic embryos, which express GFP in endothelial cells, were treated at the shield stage (6 hpf) in three groups: 0.1 µM or 0.25 µM of PDGF receptor tyrosine kinase inhibitor V (inhV, Calbiochem) [23], or DMSO for control. The IC_50_ of inhV for PDGFRs is 4∼7.6 nM. We chose to utilize inhV because it caused less overall developmental defects compared to other PDGFR inhibitors such as AG1295.

**Figure 4:**
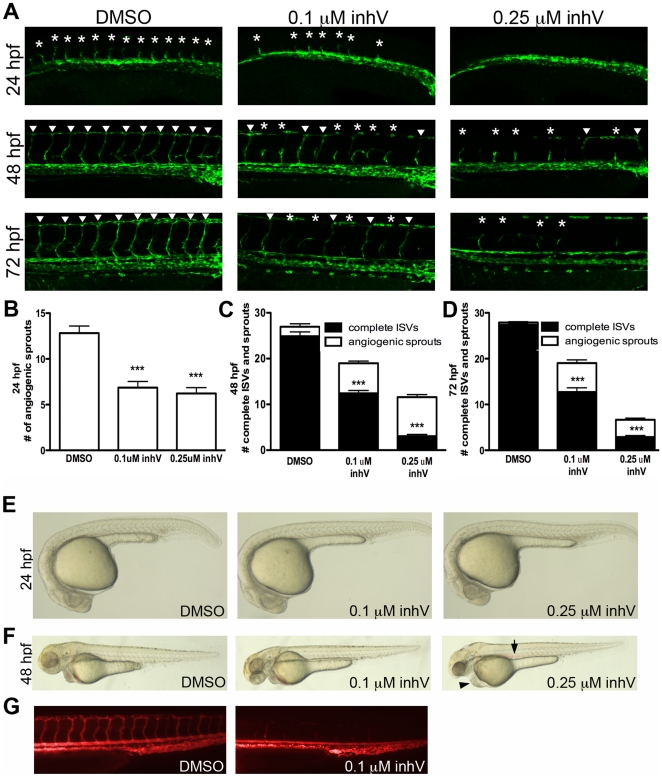
PDGFR inhibition blocked ISV formation and extension. *kdrl:GFP* embryos were treated at the shield stage (6 hpf) with DMSO alone, 0.1 µM PDGFR tyrosine kinase inhibitor V (inhV), or 0.25 µM inhV. Inhibition of PDGFRs resulted in a decrease in the number of angiogenic sprouts (asterisks in A) in the tail at 24 hpf (B) and a decrease in the number of complete ISVs (arrowheads in A) at 48 hpf (A and C) and 72 hpf (A and D). The morphology for all treatments at 24 hpf was normal (E). Embryos treated with 0.25 µM inhV began to show edema (arrowhead in F) and pooling of blood (arrow in F) at 48 hpf. Angiography at 72 hpf showed a decrease in blood circulation through the ISVs in embryos treated with inhV (G). *** indicates p<0.001. All data represent the mean +/− standard error.

Embryos treated with 0.1 µM or 0.25 µM inhV had significantly fewer angiogenic sprouts at 24 hpf than embryos treated with DMSO (Figure 4A and B, p<0.001, n = 30). When analyzed at 48 hpf, a time point where most ISVs in control embryos have fully extended dorsally to form the dorsal longitudinal anastomotic vessel (DLAV), treatment with inhV resulted in a significant reduction in the number of complete ISVs, with a greater reduction in embryos treated with 0.25 µM inhV (Figure 4A and C, p<0.001, n = 30). This deficit in ISV formation was maintained at 72 hpf (Figure 4A and D, p<0.001, n = 30). At 24 hpf there appeared to be no developmental delay of embryos treated with inhV (Figure 4E). At 48 hpf, embryos treated with 0.25 µM started to exhibit edema and loss of circulation as seen by pooling of blood in the dorsal aorta (Figure 4F). Angiography at 72 hpf showed a decrease in circulation through the ISVs of embryos treated with 0.1 µM inhV when compared to embryos treated with DMSO alone (Figure 4G). Embryos treated with 0.25 µM inhV completely lacked circulation and angiography could not be done. These data suggested that PDGFR signaling was critical for ISV angiogenesis.

### PDGFRβ2 activity was required for ISV angiogenesis in zebrafish embryos

**Figure 5:**
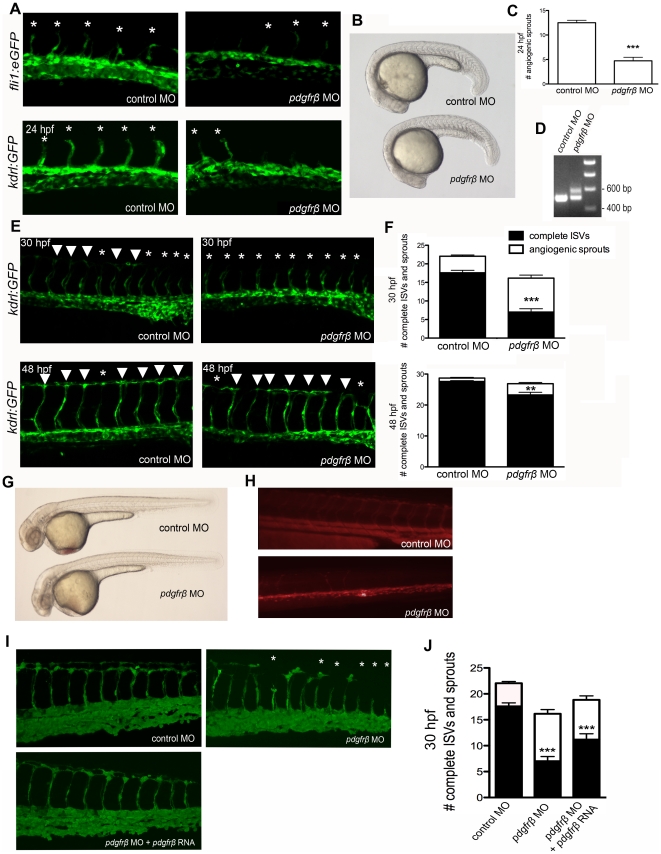
PDGFRβ2 activity was required for ISV angiogenesis in zebrafish embryos. *fli1a:eGFP* or *kdrl:GFP* embryos were injected with control morpholino oligonucleotide (MO) or *pdgfrβ2* MO at the one-cell stage and imaged at 24 hpf (A), 30 hpf and 48 hpf (E). Light images of whole embryos are shown for 24 hpf (B) and 48 hpf (G). Blocking of splicing was confirmed using RT-PCR (D). *pdgfrβ2* MO-injected embryos had significantly more ISV defects than control MO-injected embryos at 24 hpf (A and C), 30 hpf and 48 hpf (E and F). Angiography at 72 hpf showed less circulation through ISVs in *pdgfrβ2* MO-injected embryos (H). The ISV defect seen in *pdgfrβ2* MO-injected embryos was partially rescued when *pdgfrβ2* mRNA was coinjected with *pdgfrβ2* MO (I and J). Arrowheads indicate complete ISVs. Asterisks indicate angiogenic sprouts. *** indicates p<0.001, ** indicates p<0.01. All data represent the mean +/− standard error.

We further investigated the role of PDGFR signaling in ISV angiogenesis using a morpholino oligonucleotide (MO) specific to *pdgfrβ2*. We injected *fli1a:eGFP* [13] and *kdrl:GFP* [22] transgenic embryos at the one-cell stage with MOs that blocked the splicing of the eighth exon-intron junction of zebrafish *pdgfrβ2*. The disruption in splicing was confirmed by RT-PCR at 48 hpf, which indicated that approximately 30–50% of *pdgfrβ2* splicing was blocked (Figure 5D). The splicing block was targeted to affect the tyrosine kinase domain of *pdgfrβ2* while leaving the extracellular and transmembrane portions of the receptor intact. Thus, it is possible that the alternatively spliced *pdgfrβ2* was also able to function as a dominant negative form that was able to interfere with the endogenous form of the receptor. *pdgfrβ2* MO-injected embryos had a reduction in the number and extent of ISVs compared to control embryos that were injected with a control mismatch morpholino. At 24 hpf, *pdgfrβ2* MO-injected embryos had an average of 4.7 angiogenic sprouts as compared to 12.5 for control MO-injected embryos (Figure 5A and C, p<0.001, n = 18). The sprouting delay seen at 24 hpf did not result from an overall developmental delay as evidenced by no delay in somite formation (Figure 5B). When analyzed for the number of complete ISVs that extended to form the DLAV at 30 hpf, *pdgfrβ2* MO-injected embryos had an average of only 7.0 complete ISVs that extended dorsally to form the DLAV versus 17.6 complete ISVs for control MO-injected embryos (Figure 5E and F, p<0.001, n = 20). At 48 hpf, *pdgfrβ2* MO-injected embryos started to recover from the original sprouting delay with an average of 23.25 ISVs that fully extended dorsally, compared to control MO-injected embryos that had 27.6 complete ISVs (Figure 5D and E, p<0.01, n = 20). These data suggest that a decrease in MO activity between 30–48 hpf may result in ISV recovery in *pdgfrβ2* MO-injected embryos. Angiography at 72 hpf showed a decrease in circulation of ISVs of *pdgfrβ2* MO-injected embryos (Figure 5H). Overall, these results indicated that PDGFRβ2 signaling was required for ISV angiogenesis in zebrafish embryos.

To confirm that the phenotypes observed in *pdgfrβ2* MO-injected embryos were caused by loss of function in *pdgfrβ2*, we co-injected *pdgfrβ2* mRNA with *pdgfrβ2* MO. *pdgfrβ2* mRNA was not subject to the MO splice blocking and should therefore be fully functional. After the addition of *pdgfrβ2* mRNA, we observed a partial rescue of *pdgfrβ2* MO-induced defects at 30 hpf (Figure 5I and J, p<0.01, n = 18), suggesting that the defects caused by injecting the *pdgfrβ2* MO were indeed caused by a knockdown of *pdgfrβ2* activity.

**Figure 6:**
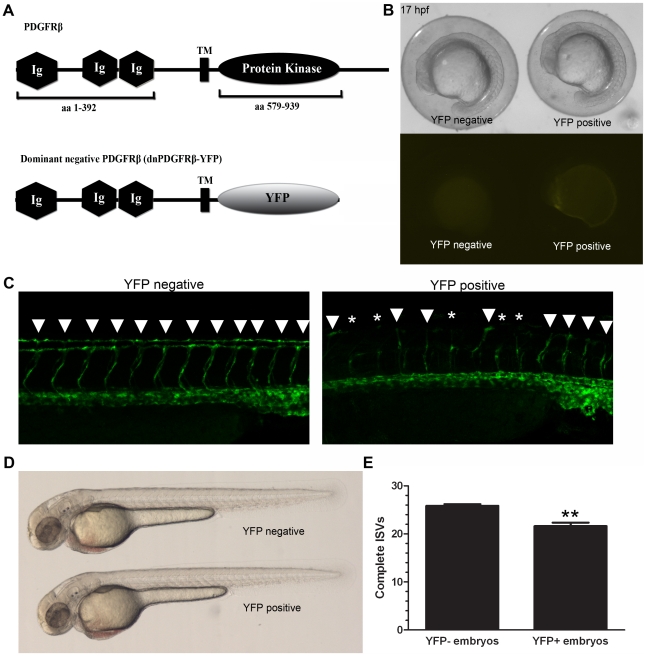
A dominant-negative PDGFRβ blocked ISV angiogenesis in zebrafish embryos. A heat shock-inducible dominant negative form of PDGFRβ was created by substituting the intracellular kinase domains of PDGFRβ with YFP (A, dnPDGFRβ-YFP). Heat shock at 10 hpf led to dnPDGFRβ-YFP expression within 7 hours after heat shock with no effect on overall morphology (B). Heat shock at 20 hpf and analysis at 48 hpf indicated an increase in ISV defects in heat shocked embryos positive for dnPDGFRβ-YFP versus heat shocked embryos negative for dnPDGFRβ-YFP (C and E, n = 23). Embryos heat shocked at 20 hpf that were positive for dnPDGFRβ-YFP at 48 hpf showed no overall morphology defects as compared to embryos heat shocked at 20 hpf negative for dnPDGFRβ-YFP (D). Arrowheads indicate complete ISVs. Asterisks indicate angiogenic sprouts. ** indicates p<0.01. All data represent the mean +/− standard error.

We also created a heat shock-inducible dominant negative PDGFRβ transgenic line to further verify the effect of PDGFRβ signaling on ISV formation. To create the dominant negative PDGFRβ (dnPDGFRβ-YFP) we replaced the intracellular tyrosine kinase domains of PDGFRβ with YFP to create a fusion protein that is inactive and has the ability to bind to endogenous PDGFRβ and prevent auto-transphosphorylation (Figure 6A). Triple transgenic *UAS*:*dnpdgfr*β*-yfp;hsp70:Gal4;fli1a:eGFP* embryos were generated by crossing the *UAS*:*dnpdgfr*β*-yfp;hsp70:Gal4* double transgenic line with the double transgenic *fli1a:eGFP;hsp70:Gal4* line to allow for visualization of the vasculature (see materials and methods for details). Embryos were heat shocked for 20 minutes in a 40°C water bath at 10 hpf or 22 hpf. When heat shocked at 10 hpf, dnPDGFRβ-YFP expression was visible within 7 hours after heat shock (Figure 6B). Heat shock at 10 hpf showed very distinct dnPDGFRβ-YFP expression localized to the notochord at 20 hpf. However, dnPDGFRβ-YFP expression was not maintained at 48 hpf and there was no effect on ISV formation (data not shown). In contrast, embryos that were heat shocked at 22 hpf expressed dnPDGFRβ-YFP throughout the tail and maintained dnPDGFRβ-YFP expression to 48 hpf and exhibited defects in ISV formation (Figure 6C and E, n = 23, p<0.0001) as shown by *fli1a:eGFP*. Expression of dnPDGFRβ-YFP resulted in no developmental delay when compared with heat-shocked embryos negative for dnPDGFRβ-YFP expression at 17 hpf or 48 hpf (Figure 6B and D).

### A critical period existed for PDGFR signaling in ISV formation

**Figure 7:**
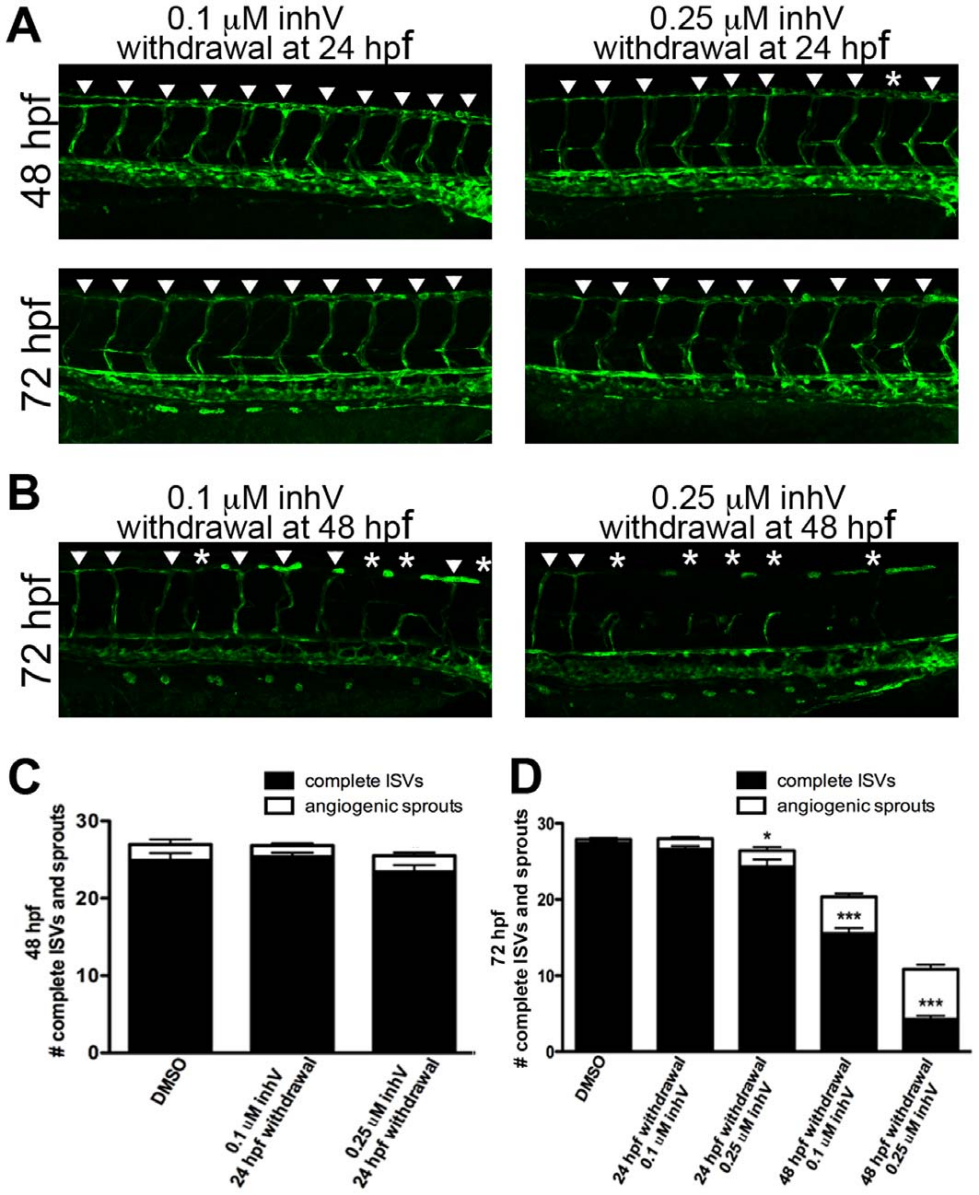
A critical period existed for PDGFR signaling in ISV formation. *kdrl:GFP* embryos were treated with inhV starting from 6 hpf. Withdrawal of PDGFR inhibition at 24 hpf resulted in full recovery of ISV number and extent within 24 hours after withdrawal (A and C). In contrast, withdrawal of inhV at 48 hpf resulted in little or no recovery of ISV formation by 72 hpf (B and D). Arrowheads indicate fully-formed ISVs. Asterisks indicate angiogenic sprouts. *** indicates p<0.001, * indicates p<0.05. All data represent the mean +/− standard error.

To determine if a critical period exists for PDGFR signaling, we treated *kdrl:GFP* embryos with inhV from 6–24 hpf or 6–48 hpf and measured the extent of ISV recovery after drug withdrawal. Withdrawal of inhV at 24 hpf (treated from 6–24 hpf with inhV) resulted in a full recovery of complete ISV formation when analyzed at 48 hpf for embryos initially treated with 0.1 µM (Figure 7A and C, p = 0.593, n = 30) and 0.25 µM inhV (Figure 7A and C, p = 0.097, n = 30) when compared to DMSO control. When analyzed at 72 hpf, embryos treated with 0.1 µM inhV continued to show no difference from control (Figure 7A and D, p = 0.363, n = 30). Embryos treated with 0.25 µM inhV from 6–24 hpf showed an almost full recovery of ISV formation at 72 hpf, but had an average of 3.09 fewer complete ISVs as compared to control (Figure 7A and D, p = 0.01, n = 30). In contrast to the recovery seen after withdrawal of inhV at 24 hpf, withdrawal at 48 hpf resulted in almost no recovery of ISV formation (Figure 7B and D, p<0.001, n = 30). The difference in the ability to recover after withdrawal at 24 hpf versus 48 hpf suggested that there was a critical period for PDGFR function in ISV angiogenesis.

### PDGFRβ signaling was mediated by PI3 kinase

**Figure 8:**
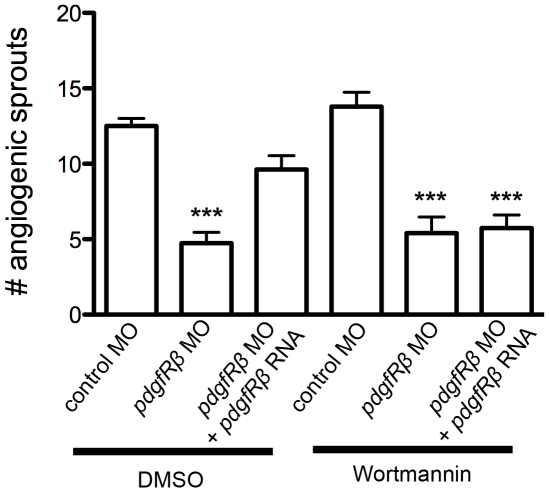
PDGFRβ2 signaling was mediated by PI3 kinase. *kdrl:GFP* embryos were injected at the one-cell stage with control morpholino oligonucleotide (MO), *pdgfrβ2* MO, or *pdgfrβ2* MO plus *pdgfrβ2* mRNA, and analyzed for angiogenic sprouting at 24 hpf in the presence of DMSO control or 100 nM of the PI3 kinase inhibitor Wortmannin. *** indicates p<0.001. All data represent the mean +/− standard error.

PI3 kinase is a known downstream effector of PDGFRβ2. It was recently shown that blocking PI3 kinase with the chemical inhibitor wortmannin causes defects in blood vessel formation in zebrafish [24]. To determine if PDGFRβ2 signaling during ISV angiogenesis in zebrafish embryos is mediated by PI3 kinase, we tested the effect of wortmannin on mRNA rescue of the *pdgfrβ2* MO phenotype. High concentrations (1 µM) of wortmannin cause defects in blood vessel formation [24]. Consistent with previous studies [24], we found that a lower dose of wortmannin (100 nM) did not affect normal ISV sprouting at 24 hpf (Figure 8). *pdgfrβ2* MO-injected embryos exhibited ISV sprouting defects at 24 hpf (Figure 5A–C). Treatment with 100 nM wortmannin did not further exacerbate the defects caused by *pdgfrβ2* MO injection (Figure 8). Co-injection of *pdgfrβ2* mRNA partially rescued the sprouting defects caused by *pdgfrβ2* MO injection when treated with DMSO as a control. In contrast, treatment with 100 nM wortmannin blocked this rescue at 24 hpf (Figure 8, p<0.001, n = 14). These data suggested that partially inhibiting PI3 kinase with 100 nM wortmannin decreased PDGF signaling throughput, and were consistent with PI3 kinase acting downstream of PDGFRβ2 signaling at 24 hpf to promote the formation of zebrafish ISVs.

## Discussion

Previous studies in mice have shown an important role for PDGFRβ2 in mural cell recruitment to the developing vasculature [4]. Here we characterize the zebrafish homolog of PDGFRβ in zebrafish embryos. We report the expression pattern of *pdgfrβ2* in the hypochord in association with the vasculature and further show the importance of PDGFRβ2 signaling in zebrafish developmental angiogenesis in the absence of mural cells. Our results demonstrate a novel role for PDGFRβ2 signaling in zebrafish developmental angiogenesis *in vivo*.

The angiogenic sprouting of zebrafish ISVs is potentially regulated by different signaling mechanisms than the *de novo* vasculogenic formation of the dorsal aorta and the arterial-venous segregation of the posterior cardinal vein [16]. Our data suggest a role for PDGF signaling in the sprouting and extension of the ISVs, as evidenced by the deficit in ISV formation after PDGFR chemical inhibition, PDGFRβ2 MO knockdown and dominant negative PDGFRβ expression. The function of PDGFRβ on ISV angiogenesis is likely to be indirect as we found that the expression of *pdgfrβ* is mainly in the hypochord and ventral somite boundary. In Xenopus, the hypochord is shown to express VEGF and is important for dorsal aorta development [25]. In zebrafish, *vegf-a* is expressed in the somites and hypochord [26] while *vegf-c* is expressed in the hypochord and dorsal aorta [20]. The expression of PDGFRβ and VEGFs in the hypochord parallels with the localized expression patterns of these genes in pericytes and mural cells in more mature vasculature.

To our knowledge, our data are the first *in vivo* findings that demonstrate the role of PDGFRβ2 in the zebrafish vasculature at the early stages of development when there are likely no mural cells present. Together, our data suggest a potential alternative PDGF-B- PDGFRβ2 signaling mechanism in immature blood vessels that is distinct from the role of PDGF-B signaling that is seen in more developed vascular networks.

## Materials and Methods

### Identification of zebrafish *pdgfrβ* and RT-PCR

To clone the zebrafish homolog of *pdgfrβ*, we utilized two pairs of primers to amplify cDNA from embryos and regenerating hearts: PDGFRβ 5′ forward: ATGAAGAGTTCGACCATCAG, PDGFRβ 5′ reverse: TCTTCCTCC ACACAGCAATG, PDGFRβ 3′ forward: TCCAGACTAATGTCACCTACAACAG, and PDGFRβ 3′ reverse: GAAGCTCTCCTCTACTTCTGGACTT. Both fragments were digested at the overlapping *Sma*I restriction site and cloned into the pGEM-T easy vector.

### Phylogenetic analysis

Sequences were selected from GenBank to represent the available major vertebrate groups (i.e., fish, amphibians, and mammals). For PDGF-B, the following sequences were selected: *Danio rerio* (ABG34342), *Xenopus laevis* (NP_001087935.1), *Xenopus tropicalis* (AAI60575.1), *Gallus gallus* (NP_989601.1), *Mus musculus* (NP_035187.2), *Rattus norvegicus* (NP_113712), *Homo sapiens* (isoform 1, NP_002599.1 and isoform 2, NP_148937.1). PDGF-A (AAH78289) was used as an outgroup to PDGF-B. For PDGFRβ, two sets of phylogenetic analyses were performed: the first analysis utilized the full length PDGFRβ2 (HM439112) and the following sequences: two Fugu isoforms: PDGFRβ1 (P79749.1) and PDGFRβ2 (AAL50567), two *H. burtoni* isoforms: PDGFRβ a (ABD48800.1) and PDGFRβ b (ABD48798.1), *Gallus gallus* (XP_001233830.1), *Mus musculus* (NP_032835.1), *Rattus norvegicus* (NP_113713.1), *Homo sapiens* (NP_002600.1 and isoform CRA_a, EAW61746.1). The second analysis included a 64 amino acid residue fragment of PDGFRβ1 from *D. rerio* (AAN02896), with the sequences of other species edited to correspond to the 64 aa fragment. The sequences for each set of genes were aligned in Clustal X [27] and then analyzed for the most appropriate model of protein evolution using ProtTest [28]. For PDGF-B, the phylogenetic relationships among sequences were estimated using Bayesian analysis with MrBayes v3.0.4b [29] with the Jones-Taylor-Thorten [30] model of protein evolution with gamma distributed rates, 10,000,000 generations with 4 chains and a burn-in of 10% of the generations before trees were sampled. A majority rule consensus tree for this analysis was constructed in PAUP 4.0B [31]. For the two PDGFRβ analyses, the phylogenetic relationships among sequences were estimated using minimum evolution with the Jones-Taylor-Thorten [30] model of protein evolution with gamma-distributed rates, and 1,000 bootstrap replications. A consensus tree for each analysis was constructed.

### Zebrafish husbandry, chemical inhibition, and morpholino injection

Zebrafish were maintained on a 14 hr light/dark cycle at 28.5°C. All experiments involving zebrafish were performed according to the National Institute of Health guidelines and have been approved by the Institutional Animal Care and Use Committee of the Childrens Hospital Los Angeles. Embryos were collected and raised in E3 media according to standard protocol [32]. All embryos were treated with 0.2 mM 1-phenyl-2-thiourea (PTU) at 22 hpf to prevent pigmentation. A stock solution of 1 mM PDGF receptor tyrosine kinase inhibitor V (inhV, Calbiochem) dissolved in DMSO was used for all chemical inhibitor experiments. Embryos were treated at 6 hpf with DMSO alone, 0.1 µM or 0.25 µM inhV in E3 media and protected from the light, as inhV is light sensitive. E3 media, PTU, and inhV were refreshed daily. In the cases of 24 hpf and 48 hpf inhV withdrawal, media was replaced at the indicated time with E3 media and PTU only.

Embryos were injected in E2 media at the one-cell stage with 3.5 ng morpholino oligonucleotides (MO). MOs were obtained from Gene Tools (Philomath, OR) with the following oligonucleotide sequences: pdgfrβ MO, ACA GGA ACT GAA GTC ACT GAC CTT C; pdgfrβ control MO, ACA CGA ACT GAA CTC AGT GAC GTT C. The control MO had five nucleotides that differed from the experimental MO.

### *In situ* hybridization

Embryos were fixed in 4% paraformaldehyde at 4°C for 16 hours and then transferred to methanol at −20°C. Embryos were then treated as previously described [33].

### Ultrastructural examination

Zebrafish embryos were fixed with 2% glutaraldehyde in phosphate buffer, post-fixed with 1% osmium, and embedded in Epon. The areas of interest were discussed and confirmed with light microscope in longitudinal sections stained with toluidine blue solution. The ultra-thin sections were cut onto one-hole grids, stained with uranyl and lead, and examined with Morgagni 268.

### Generation of dnPDGFRβ-YFP fish

To achieve temporal control over expression of the dominant negative receptor, we utilized a GAL4-UAS system [34] and generated *UAS*:*dnpdgfr*β*-yfp* transgenic fish lines. We then crossed the *UAS*:*dnpdgfr*β*-yfp* transgenic fish *to hsp70:Gal4* fish to obtain a double transgenic line (*UAS*:*dnpdgfr*β*-yfp;hsp70:Gal4*) [35], [36]. The heat shock protein 70 (*hsp70*) promoter allowed for temporal control of expression of Gal4. The double transgenic *UAS*:*dnpdgfr*β*-yfp;hsp70:Gal4* line was then crossed with a second double transgenic *fli1a:eGFP;hsp70:Gal4* line to generate triple transgenic *UAS*:*dnpdgfr*β*-yfp;hsp70:Gal4;fli1a:eGFP* embryos to be used for the experiment. Embryos obtained from this crossing were then heat shocked for 20 minutes in a 40°C water bath at 10 hpf or 20 hpf. Embryos were then sorted for expression of YFP. 40 embryos each from the YFP-positive and the YFP-negative embryos were selected for their normal morphology at 48 hpf as heat shock alone caused some overall morphology defects in approximately 10–20% of embryos. These embryos were selected under normal light illumination to prevent biased selection under GFP fluorescence. From these 40 embryos, the GFP-positive embryos (n = 23 for each of the YFP-positive and YFP-negative groups) were analyzed for full ISV extension.
